# Enhancement of recombination process using silver and graphene quantum dot embedded intermediate layer for efficient organic tandem cells

**DOI:** 10.1038/srep30327

**Published:** 2016-07-25

**Authors:** Nhu Thuy Ho, Huynh Ngoc Tien, Se-Joeng Jang, Velusamy Senthilkumar, Yun Chang Park, Shinuk Cho, Yong Soo Kim

**Affiliations:** 1 Department of Physics and Energy Harvest Storage Research Center, University of Ulsan, Ulsan 44610, S. Korea; 2 Department of Chemical Engineering, University of South Carolina, Columbia, South Carolina 29208, USA; 3 Measurement and Analysis Division, National Nanofab Center, Daejeon 34141, S. Korea

## Abstract

High performance of organic tandem solar cell is largely dependent on transparent and conductive intermediate layer (IML). The current work reports the design and fabrication of an IML using a simple solution process. The efficiency of a homo-tandem device with poly(3-hexylthiophene):phenyl-C61-butyric acid methyl ester as an active layer and poly(3,4-ethylenedioxythiophene):poly(styrenesulfonate)/poly(ethylenimine) as an IML was initially found to be 3.40%. Further enhancement of the cell efficiency was achieved using silver nanoparticles (Ag-NPs) of different sizes and graphene quantum dot embedded IML. A maximum efficiency of 4.03% was achieved using 7 nm Ag-NPs that contribute to a better recombination process. Also, the performance of the tandem cell was solely based on the electrical improvements indicated by the current - voltage measurements, external quantum efficiency and impedance analysis. The use of Ag-NPs in the IML has been shown to lengthen the life time of electron-hole pairs in the device. This study thus paves way to develop such efficient IMLs for more efficient tandem solar cells.

Organic polymer solar cell is an emerging photovoltaics (PVs) technology with promising properties due to its portability, ultralight, flexibility, transparency, cost-effectiveness and large-area manufacturing compatibility^1^,^2^,^3^. Over the decades, intensive research has focused on organic photovoltaic cells (OPVs) for development of the cell efficiencies in various approaches such as the combination of photoactive material design^4^,^5^,^6^,^7^, morphology control^8^, and interface engineering^9^,^10^,^11^. Consequently, the efficiency of OPVs has been boosted up to ~10%^12^. However, the spectrally limited absorption nature of the organic donor materials and low charge carrier mobility results in relatively poor short-circuit current density (*J*_sc_) of PVs^1^,^2^,^3^,^4^,^5^. An increase in the *J*_sc_ of OPVs can be easily achieved by the use of ternary or multicomponent donor-acceptor systems that broaden the absorption spectra of the organic semiconductors^13^,^14^. Another drawback limiting the overall cell efficiency of OPVs is low open-circuit voltage (*V*_oc_). Recent studies have reported on the use of semiconductors with high dielectric constant to reduce the thermalization loss to a certain extent^15^.

To overcome these issues, another approach has been proposed, known as the tandem cell concept, which stacks two or more cells with complementary absorption spectra in series or parallel connections. The tandem cell structure was found to be a solution to both the *J*_sc_ related absorption losses as well as *V*_oc_ related thermalization issues^16^,^17^. Using this concept, numerous organic tandem solar cells were designed with efficiency up to 11%^18^. For a tandem solar cell, choosing an intermediate layer (IML) that connects the two sub-cells in a series-connected sequence of a hole-transporting layer (HTL) and an electron-transporting layer (ETL) is a critical consideration for the device performance. To realize high performance tandem cells, an efficient and reliable IML is mandatory^19^. Charge recombination process at this layer should be efficient to maximize the *V*_oc_ and fill factor (FF).

The commonly used IML layers in the organic tandem solar cells that comprised of both high and low work-function components, such as LiF/Al/Au/PEDOT:PSS^20^, LiF/Al/MoO_3_^21^, MoO_3_/Al/ZnO^22^, MoO_3_/Ag/Al/Ca^23^, or Al/TiO_2_/PEDOT:PSS^24^ require high vacuum for the synthesis of certain IML layers. The recently reported solution-processed IML layers containing PEDOT:PSS/ZnO^25^ for inverted tandem cells, and ZnO/PEDOT:PSS^26^ and TiO_X_/PEDOT:PSS^27^ for conventional tandem cells have been a success to a certain limit. Moreover, all solution-processed IMLs are highly desirable for large-area solar cell fabrication. In addition, the use of crystalline inorganic materials results in less mechanical flexibility. Also, the difference between the work function of the widely used HTL of the PEDOT:PSS and ETL of metal oxides is less than approximately 0.6 eV, thus limiting the use of photoactive materials for the assembly of tandem solar cells. Hence, interfacial engineering problems related to IMLs is a crucial factor in obtaining high efficiency tandem solar cells.

Concerning the above issues, we report on a study the design and fabrication of an all solution-processed tandem cell structure with poly (3-hexylthiophene): phenyl-C61-butyric acid methyl ester (P3HT:PCBM) as an active layer in both front and rear sub-cells and PEDOT:PSS/PEI as an IML. Poly(3,4-ethylenedioxythiophene):poly(styrenesulfonate)/poly(ethylenimine) (PEI) was recently introduced as a powerful ETL for its adjustable work function, formation of tunnel junction, and a strong connection with the PEDOT:PSS material due to deprotonated sulfonic acid^28^,^29^. Also, to further enhance the recombination process, semitransparent and highly conductive silver nanoparticles (Ag-NPs) and graphene quantum dots (G-QDs) were incorporated between the layers of IML for efficient collection of the holes and electrons from the sub-cells with no potential loss. A systematic study on the different sizes of Ag-NPs was probed was performed. Intensity modulated photocurrent/voltage spectroscopic (IMPS/IMVS) studies were then performed to compare the recombination time and transit time of the tandem solar cells without and with Ag-NPs in the IML.

## Results

**Figure 1:**
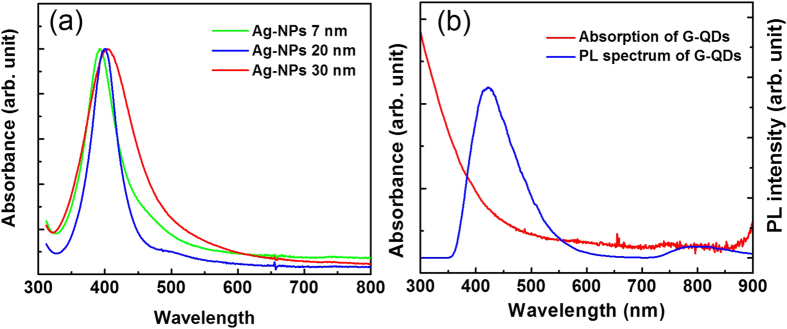
(**a**) UV-Vis absorption spectra of Ag-NPs, (**b**) UV-Vis absorption (red curve) and PL spectrum (blue curve) of G-QDs.

The optical properties of the as-synthesized Ag-NPs and G-QDs dispersed in ethanol were examined. Figure 1(a) shows the absorption results of the three different sizes of Ag-NPs analyzed in the spectral range of 300 to 800 nm. The sharp peak in the absorption spectra of around 400 nm indicates that the prepared Ag-NPs are uniform in size distribution. The absorption peak slightly red shifted towards the higher wavelength with an increase in particle size. An absorption spectrum of metal NPs may be described as a result of the intra-band excitations of conduction electrons from the lowest energy state to higher energy state within the conduction band of metal NPs^30^,^31^. In addition, photoluminescence (PL) studies were performed for these Ag-NPs at room temperature (RT). However, no luminescence was observed. Although some studies have reported on the PL on silver clusters in a certain matrix at a very low temperature^32^, it should be noted that systematic works on the PL of Ag-NPs at RT were very weak^33^. The absorption spectrum of G-QDs clearly showed that there was no absorption in the wavelength range of 300–800 nm, while the PL showed a peak at 423 nm (Fig. 1(b)). It is important to notice that the peak of PL shifted slightly according to the concentrations of G-QDs. This might be due to the stacking of the G-QDs at a higher concentration.

**Figure 2:**
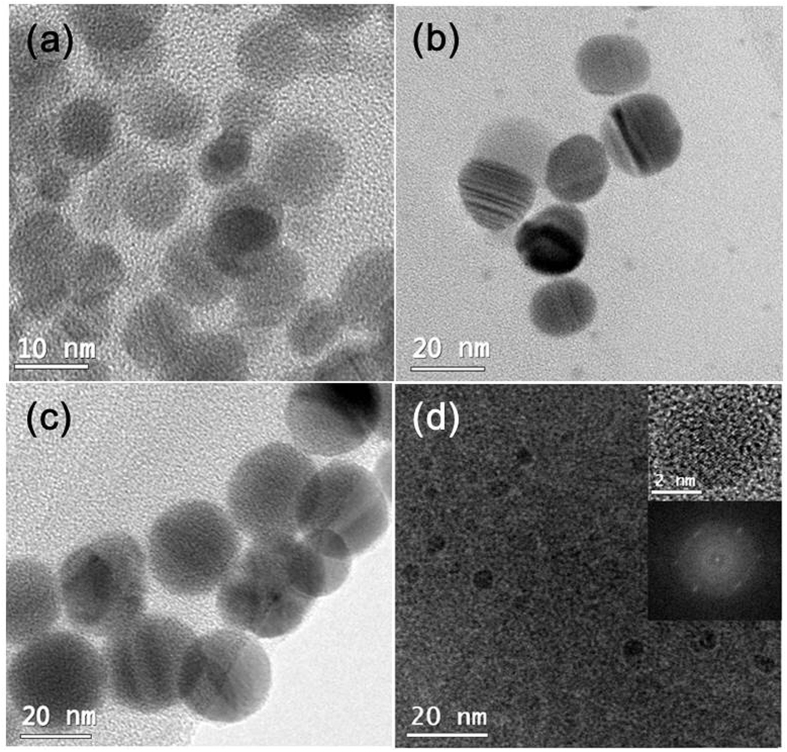
(**a**) TEM image of Ag-NPs of 10 nm, (**b**) 20 nm, (**c**) 30 nm and (**d**) G-QDs. Inserted in (**d**) high resolution images and its diffraction pattern.

The transmission electron microscopy (TEM) images of Ag-NPs synthesized with three different sizes using the Lee-Meisel method are shown in Fig. 2(a–c). The average particle sizes of Ag-NPs, which were estimated from the TEM images, were 7, 20, and 30 nm, respectively. To confirm the uniform size distribution of the Ag-NPs, these NPs were spin-coated on Si substrate. The AFM measurements for these samples clearly indicated that the Ag-NPs were well distributed on the whole substrate (see SI, Figure S1 and Figure S2). The low-magnification TEM image of G-QDs is shown in Fig. 2(d). The average particle size was found to be around 4 nm. In addition, these characterized Ag-NPs and G-QDs were embedded in IML of PEDOT: PSS/PEI to improve the efficiency of the organic tandem solar cell.

**Figure 3:**
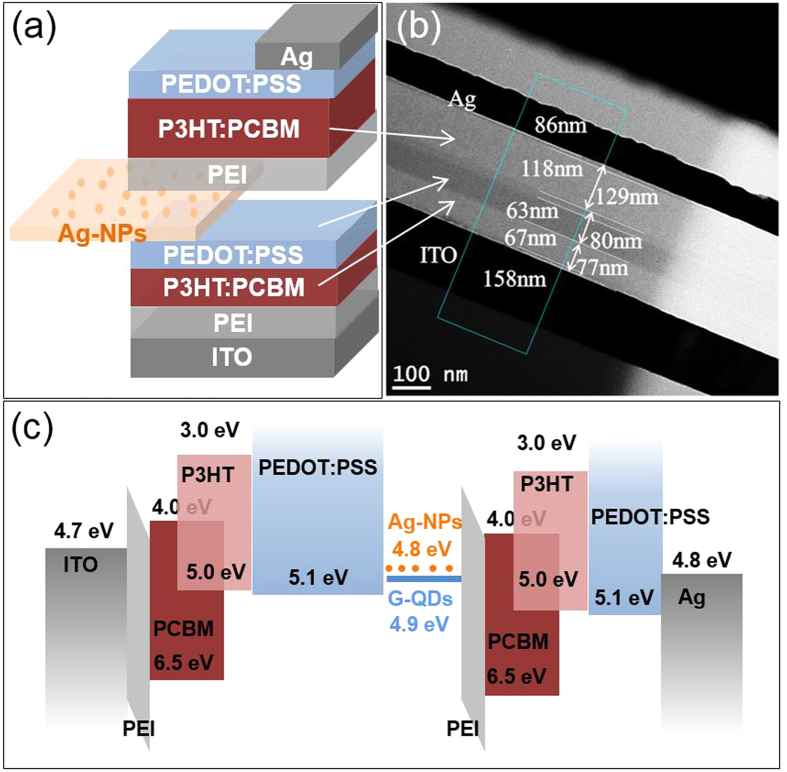
(**a**) Schematic of a tandem organic solar cell based on P3HT:PCBM active layer, (**b**) cross-sectional TEM image of the device (FIB method), and (**c**) band alignment of the device.

The schematic diagram, its cross-sectional TEM image and band alignment of the proposed device structure are as shown in Fig. 3(a–c). The step profile of each layer in Fig. 3(b) was clearly observed by focused ion beam-TEM measurements. The device consisted of two combined sub-cells, the front and rear cell connected in a series by Ag-NPs embedded IML of PEDOT: PSS/PEI. The PEDOT: PSS acted as an HTL whereas the PEI layer served as an ETL. In addition, a thick PEDOT: PSS (63 nm) layer was used on the top of the front cell to protect the front cell layer from damage during fabrication of the rear cell.

**Figure 4:**
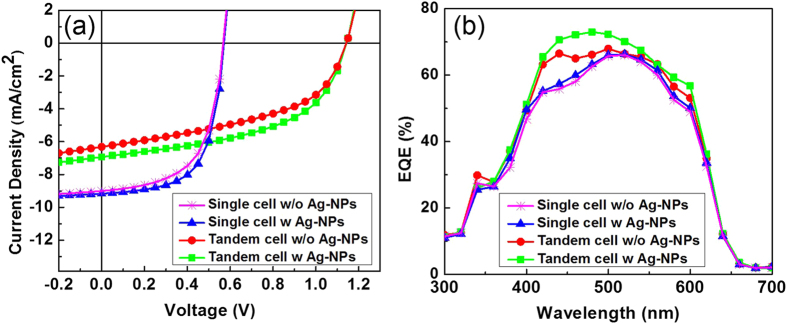
(**a**) Current density-voltage characteristic and (**b**) External quantum efficiency of single and tandem OSC devices.

To examine the influence of Ag-NPs, current density–voltage (*J-V*) measurements were performed for the Ag-NPs inserted tandem device under 1.5 AM illumination as shown in Fig. 4(a). For better understanding, the single and tandem cells, with and without Ag-NPs were measured and the results are shown in a Table 1. The maximum cell efficiency and the *V*_oc_ of the tandem cell without Ag-NPs reached to 3.40% and 1.15 V, respectively. These results clearly indicated that PEDOT:PSS and PEI can also serve as a good IML even without Ag-NPs. For the tandem device with Ag-NPs there was a significant improvement in FF and *J*_sc_, which increased from 47% to 51% and 6.31 to 6.91 mA/cm^2^, respectively. The enhancement ratio of the two values was around 10%. Consequently, the photocell convergence efficiency (PCE) was thus increased from 3.40% to 4.03%. The shunt resistance (*R*_sh_) increased from 4280 to 4990 Ω.cm^2^, whereas the series resistance (*R*_*s*_) decreased from 41.8 to 34.7 Ω.cm^2^. For further confirmation, a single cell was constructed with Ag-NPs (ITO/PEDOT:PSS/Ag-NPs/PEI/P3HT:PCBM/PEDOT:PSS/Ag), and its parameters were compared to those of the inverted single cell without Ag-NPs. From these results, only the FF of the single cell with Ag-NPs increased, thus the cell’s FF value was directly affected by the photocell’s series and shunt resistances values. Hence, an increase in the *R*_sh_ and a decrease in the *R*_s_ lead to a higher FF, thus resulting in an enhancement of efficiency to 18% in the Ag-NPs inserted tandem device, which can be attributed to the better electrical properties of the device structure.

Further, we investigated the different sizes of Ag-NPs embedded in the IML of the same tandem cell structure and the results are presented in supporting information (see Figure S3). On increasing the particle size, the *J*_sc_ value increased from 6.9 to7.8 mA/cm^2^ due to the enhancement of plasmonic effects. However, the *V*_oc_ value showed a drastic decrease and the *J*-*V* curve changed to S-shape. It could be possibly due to the lesser thickness of the PEI layer (10 nm) which cannot uniformly cover large sized Ag-NPs. This in turn leads to direct contact of the Ag-NPs with the active material. Hence, an IML layer is very important in a tandem cell as it connects the two sub-cells in a series, and is directly related to the *V*_oc_ values. This is the reason that smaller sized Ag-NPs (7 nm) inserted into tandem cells show higher efficiency when compared to the larger sizes. The maximum *V*_oc_ value was obtained at a lower concentration of Ag-NPs at 0.01 mg/ml (see Figure S4).

## Discussion

A similar approach was applied to tandem OSC by using semiconductor G-QDs instead of Ag-NPs. The obtained results are shown in Table 2. However, the cell efficiency of the G-QDs inserted tandem cell was slightly lower than that of the Ag-NPs inserted tandem cell. A G-QDs spin-coated at a concentration of 5 μg/ml showed slight improvement in FF and *J*_sc_ values. Thus, the cell efficiency increased from 3.40 to 3.72%. The lower efficiency with G-QDs when compared to Ag-NPs may be due to the dangling bond of passivated hydrogen of PEI during the fabricating process^34^. In addition, a thicker layer of G-QDs leads to a decrease in device efficiency due to lower conductivity between localized quantum dots.

Further, EQE of the optimized tandem cell with a 10 nm Ag-NPs inserted device was measured. The comparative results of EQE for single as well as tandem cell, with and without Ag-NPs are as shown in Fig. 4(b). The EQE of the tandem cell was estimated by multiplying the absorption of the tandem cell (under reflection mode) by the internal quantum efficiency (IQE) of the single-junction cell^35^. Noticeably, there was no significant change in the single cell, with and without Ag-NPs in the wavelength range of 300–800 nm. This may be due to the light absorption and plasmonic effect of Ag-NPs that are limited by the use of small sized Ag-NPs as well as low concentration. In the previous reports on use of various size controlled Ag-NPs in organic solar cells^36^,^37^, the plasmonic forward scattering effect was tuned by the size of Ag-NPs. According to these studies, Ag-NPs of ~60 nm in size and at optimized concentration showed maximum PCE enhancement. The small sized Ag-NPs have been demonstrated to show a very weak plasmonic effect. Thus, in the current study Ag-NPs of 7 nm did not influence the device performance based on its plasmonic effect. Based on these results it was concluded that the enhancement of EQE in the tandem device was not related to an optical effect.

Previous work on organic tandem solar cells demonstrated that insertion of a small-size metal cluster between the sub-cells has two major functions: (1) serving as recombination centers for unpaired charges that are photo-generated in the device’s interior and (2) inducing a strong near-field to enhance the absorption of the active layer^38^. It can be presumed that insertion of Ag-NPs induces “dopant” levels between PEDOT:PSS and PEI, and introduces new surface recombination centers inside the IML.

**Figure 5:**
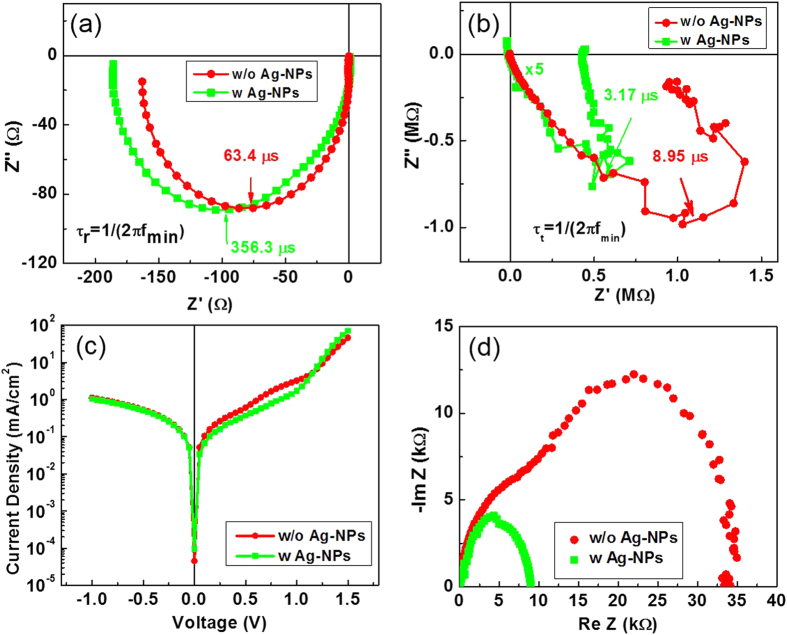
(**a**) IMVS at open-circuit condition, (**b**) IMPS at *V*_oc_, (**c**) semi-log plot of dark current density-voltage, and (**d**) Nyquist plot of the impedance of tandem OSC according to with and without Ag-NPs.

To examine the device properties in detail, light intensity modulated photocurrent/voltage spectroscopy^39^ was performed on the device, with and without Ag-NPs. The IMVS and IMPS results obtained under illumination of a red LED light (635 nm) are shown in Fig. 5(a,b). The calculated recombination time (*τ*_*r*_, from IMVS) and transit time (*τ*_*t*_, from IMPS) for the tandem cells without Ag-NPs were 63.4 μs and 8.95 μs, respectively. For the tandem cell with the Ag-NPs layer, *τ*_*r*_ and *τ*_*t*_ were calculated as 356.3 μs and 3.17 μs, respectively. From the results, the *τ*_*t*_ of the tandem cell with Ag-NPs was not significantly affected by Ag-NPs, whereas the life-time of the electron-hole pair was significantly increase. It can be explained by the additional Ag-NP layer that builds up effective recombination sites in IML thus leading to less accumulation of photo-excited charges at the active interface. Figure 5(c,d) shows the dark *J*-*V* curve and impedance data of the tandem cell, with and without Ag-NPs. The *J*-*V* characteristics are primarily determined by *R*_sh_ at a low voltage region (0–1 V), and by *R*_s_ at a higher voltage region (1–1.5 V). Here, the tandem cell showed obvious diode properties. A shunt current approaching zero in the lower voltage region along with a steep slope in a higher voltage region indicates that the resistance of the multilayer structure has been modified by introduction of the Ag-NPs layer. This is consistent with the results for calculated resistance obtained from *J*-*V* measurement under bright condition. In addition, as shown in Fig. 5(d), reduction of whole tandem system impedance from 35 kΩ back to 8 kΩ confirmed the electrical domination of modified IML on performance parameters. These results showed that the recombination nature of IML was effectively enhanced by the insertion of the Ag-NP layer. Yet, considering the capability of application of such different active material, the proposed architecture stands a chance of improving the Ohmic contact of IML by a simple solution process.

To summarize, we have studied an efficient IML embedded with Ag-NPs and G-QDs to enhance the *J*_sc_ and FF of organic tandem solar cells. The impedance of the whole device was decreased by 77% and shunt resistance increased by 15% in the presence of the Ag-NPs. Consequently, efficiency of P3HT:PCBM based homo-tandem cell was enhanced from 3.40% to 4.03%. To the best of our knowledge, this is one of the highest efficiency P3HT:PCBM based solar devices. The existence of “dopant” levels between PEDOT:PSS and PEI and inducing surface recombination centers inside the IML are attributed to causing noticeable change in electrical properties of the device. A better Ohmic contact between two sub-cells is predicted to lengthen the life-time of photo-generated electron-hole pairs by reducing accumulated charges surrounding IML. The simple but effective modification of a commonly used IML structure shows its capability of application to various other polymer materials and open up opportunities for reaching highly efficient tandem solar cells.

## Methods

### Synthesis of Ag nano-particles

Synthesis of Ag-NPs was performed using the Lee-Meisel method^40^. The chemicals silver nitrate (AgNO_3_), sodium borohydride (NaBH_4_), and trisodium citrate dihydrate (C_6_H_5_Na_3_O_7_·2H_2_O) were purchased from Shanghai Sinopharm Chemical Reagent Co., Ltd. (China) and used as received. The glassware was cleaned using a solution of HCl:HNO_3_ in a 3:1 ratio (v/v) and rinsed with deionized (DI) water prior to the experiments. Following addition 20 mL of 1% (w/v) citrate solution and 75 mL of DI water to a round bottom flask, the mixture was heated to 70 °C for 15 min. Then, 1.7 mL of 1% (w/v) AgNO_3_ solution was added to the mixture followed by rapid addition of 2 mL of 0.1% (w/v) freshly prepared NaBH_4_ solution. The reaction solution was kept at 70 °C under vigorous stirring for 1 h and cooled to room temperature. The final volume was made up to 100 ml with DI water. The resulting Ag-NPs solution was centrifuged at 10000 rpm for 30 minutes. Stepwise growth was used to obtain a larger size of Ag-NPs; 2 mL of 1% citrate solution was mixed with 75 mL of DI water and then brought to boiling using a heating mantle for 15 min. Next, 10.0 mL of starter seed solution was added while vigorous stirring, followed by addition of 1.7 mL of 1% AgNO_3_ solution. Vigorous mechanical stirring was continued for 1 h. In the next step, 2 mL of 1% citrate solution and 1.7 mL of 1% AgNO_3_ solution were dropped into the reaction solution. Reflux with vigorous stirring continued for an additional one hour, and then the reaction solution was cooled to room temperature. Water was added to bring the volume up to 100 mL. The size of resulting Ag-NPs was approximately 20 nm. To obtain 30 nm Ag-NPs, one more step was added using the same operation. Resulting Ag-NPs solutions then were centrifuged at 10000 rpm.

### Synthesis of graphene quantum dots

G-QDs were synthesized by carbonization of citric acid (CA, 99%, Sigma-Aldrich) with ammonia through hydrothermal treatment^41^; 80 ml of a CA aqueous solution (100 mg/ml) and a 20 ml (30%) ammonia aqueous solution and were heated at 180 °C in a Teflon-lined autoclave for 24 h. After cooling to room temperature, pH of the light yellow G-QDs solution was adjusted to 8 by additional of NaOH solution (1 mg/ml) dropwise. The G-QDs solution was then dialyzed in dialysis tubing (3000 Da, Spectrum Lab. Inc.) against DI water for 4 h for removal of impurities and excess ammonia. The aqueous suspension of G-QDs was then centrifuged at 10000 rpm for removal of any conglomerate.

### Single junction device fabrication

The chemicals, PEI (Aldrich, 50 wt% in H_2_O), P3HT (Merck), PCBM (EMS index), PEDOT:PSS AI4083 and PEDOT:PSS Clevios P (Heraeus) were used as received. The pre-cleaned indium tin oxide (ITO) substrates were first treated with UV-ozone for 1 hour. PEI solution (0.1 wt% diluted in iso-propanol) was spin-coated at 5000 rpm on the ITO substrates and dried at 100 °C. P3HT:PCBM (1:1) was dissolved in 1,2-dichlorobenzene at a concentration of 13.1 mg/ml and was then spin coated on the PEI surface. Prior to the spin-coating of the active layer, the solution was heated at a range of 60–80 °C for 2 h to improve the solubility, and then the diluted PEDOT:PSS (Al 4083) in iso-propyl alcohol (IPA) (1:6) was spin coated on the active layer. Finally, the samples were transferred into the evaporation chamber for fabrication of the Ag electrode with a device area of 0.13 cm^2^.

### Tandem devices fabrication

The front cell was constructed according to the single junction procedure with ITO/PEI/P3HT:PCBM, followed by spin coating a layer of diluted PEDOT:PSS in iso-propyl alcohol (1:7). This PEDOT:PSS layer was used to modify the surface of the active layer. Another layer of PEDOT:PSS was spin coated at 5000 rpm. The sample was annealed at 140 °C for 10 minutes and Ag-NPs solution (0.01 mg/ml in ethanol) was spin coated at 2000 rpm on the surface of the PEDOT:PSS layer. Subsequently, the rear cell was also fabricated with PEI/P3HT:PCBM. Thicknesses of the active layer were controlled by the speed of spin coating process. After a while, the samples were spin coated with diluted PEDOT:PSS in IPA (1:6) and transferred into the evaporation chamber. Finally, an Ag metal electrode of 100 nm thickness was deposited through a shadow mask with a device area of 0.13 cm^2^.

### Device Characterization

Atomic force microscopy (AFM; Dimension 3100, Veeco) and NIR-UV-VIS spectrometer (Carry 500, Varian) were used to observe the size of Ag-NPs. IMVS and IMPS measurement were carried out using impedance analyzer (AviumStat, IVIUM Tech), which measure the optoelectronic frequency response in the frequency range of 1 M Hz down to 1 Hz with a LED light source (IVIUM Tech., ModuLight) in the air and room temperature. The LED (λ = 635 nm) provided both the DC and AC components of the illumination, where the modulation depth of the AC component superimposed on the DC light was 10%. The light intensity was 0.86 mW/cm^2^, which was measured silicon photodiode. IMPS and IMVS were obtained under short-circuit and open-circuit condition, respectably. *J-V* characteristics of photovoltaic cells were taken using a Keithley 2400 source measure unit under a simulated AM1.5G spectrum, with an Oriel 9600 solar simulator. During the measurement, each finger was absolutely isolated by scratching surrounding films around the devices to avoid parasitic current. External quantum efficiencies (EQE) were measured using the PV measurement system in ambient pressure. The total absorption of the device was evaluated by measuring reflection (*R*) of the device and the absolute absorption of the device was calculated by (100−*R*)%. The reflection was performed using a NIR-UV-Vis spectrometer with a reflection accessory.

## Additional Information

**How to cite this article**: Ho, N. T. *et al*. Enhancement of recombination process using silver and graphene quantum dot embedded intermediate layer for efficient organic tandem cells. *Sci. Rep.* **6**, 30327; doi: 10.1038/srep30327 (2016).

## Supplementary Material

Supplementary Information

## Figures and Tables

**Table 1:** Device parameters of tandem OSC with and without Ag-NPs.

Sample	*J*_sc_ (mA/cm^2^)	*V*_oc_ (V)	FF (%)	*η*(%)	*R*_s_ (Ω·cm^2^)	*R*_sh_ (kΩ·cm^2^)
Single cell w/o Ag-NPs	8.93	0.58	0.60	3.10	9.69	4.13
Single cell w Ag-NPs	9.00	0.58	0.62	3.23	10.3	5.32
Tandem cell w/o Ag-NPs	6.31	1.15	0.47	3.40	41.8	4.28
Tandem cell w Ag-NPs	6.91	1.15	0.51	4.03	34.7	4.99

**Table 2:** Device parameters of tandem OSC with and without G-QDs.

Sample	*J*_sc_ (mA/cm^2^)	*V*_oc_ (V)	FF (%)	*η*(%)	*R*_s_ (Ω·cm^2^)	*R*_sh_ (kΩ·cm^2^)
Single cell w/o G-QDs	8.46	0.58	0.60	2.94	10.9	4.51
Single cell w G-QDs	8.95	0.58	0.61	3.15	10.6	5.07
Tandem cell w/o G-QDs	6.28	1.15	0.47	3.38	50	4.17
Tandem cell w G-QDs	6.61	1.15	0.49	3.72	45	4.80
