# Supplementary material for: Enhancement of recombination process using silver and graphene quantum dot embedded intermediate layer for efficient organic tandem cells
Source: Sci Rep. 2016 Jul 25;6:30327. doi: 10.1038/srep30327 (PMC4958952; doi:10.1038/srep30327)
Supplement: Supplementary Information [file srep30327-s1.doc]

**Supplementary Information**

**Enhancement of recombination process using silver and graphene quantum dot embedded Intermediate Layer for Efficient Organic Tandem Cells**

N. T. Ho^1^, H. N. Tien^2^, S.-J. Jang^1^, V. Senthilkumar^1^, Y. C. Park^3^, S. Cho^1^, and Y. S. Kim^1*^

*^1^Department of Physics and Energy Harvest Storage Research Center, University of Ulsan, Ulsan 44610, S. Korea, ^2^Department of Chemical Engineering, University of South Carolina, Coumbia, South Carolina 29208, USA, ^3^Measurement and Analysis Division, National Nanofab Center, Daejeon 34141, South Korea*

*[yskim2@ulsan.ac.kr](mailto:yskim2@ulsan.ac.kr) (Y. S. Kim)

**I. Size distribution of Ag-NPs and G-QDs**

**
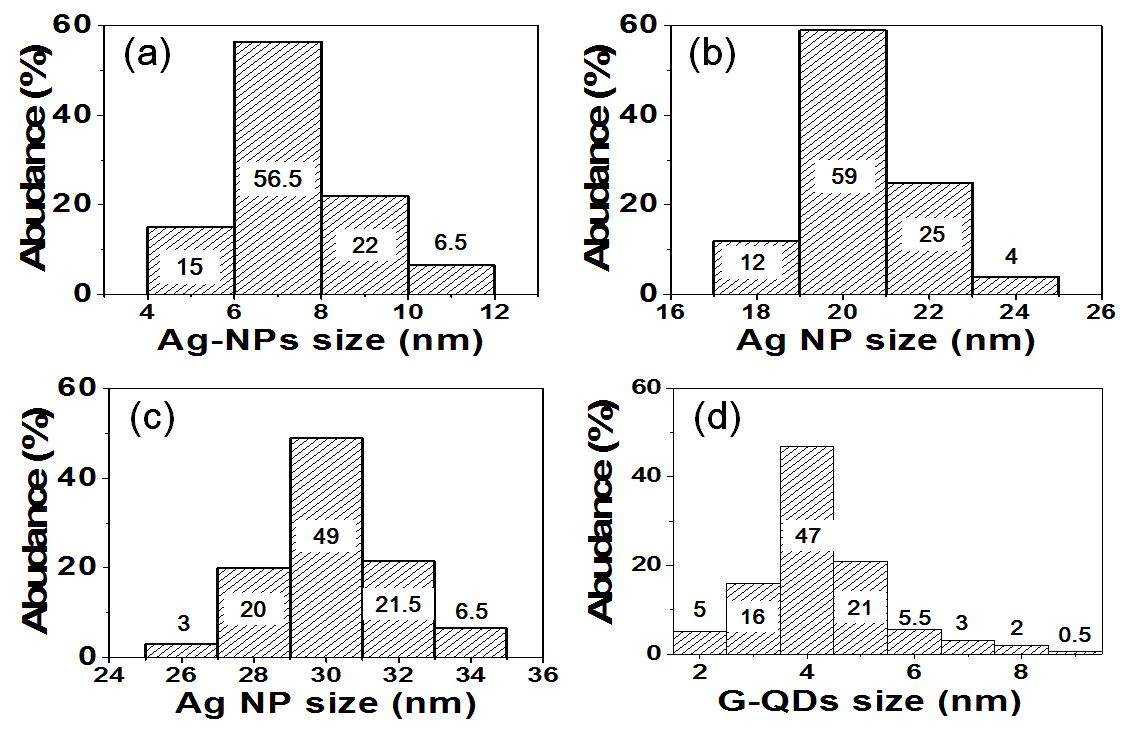
**

**Fig. S1:** Diagram for size distribution of 10nm (a), 20 nm (b), 30 nm (c) Ag-NPs and (d) G-QDs


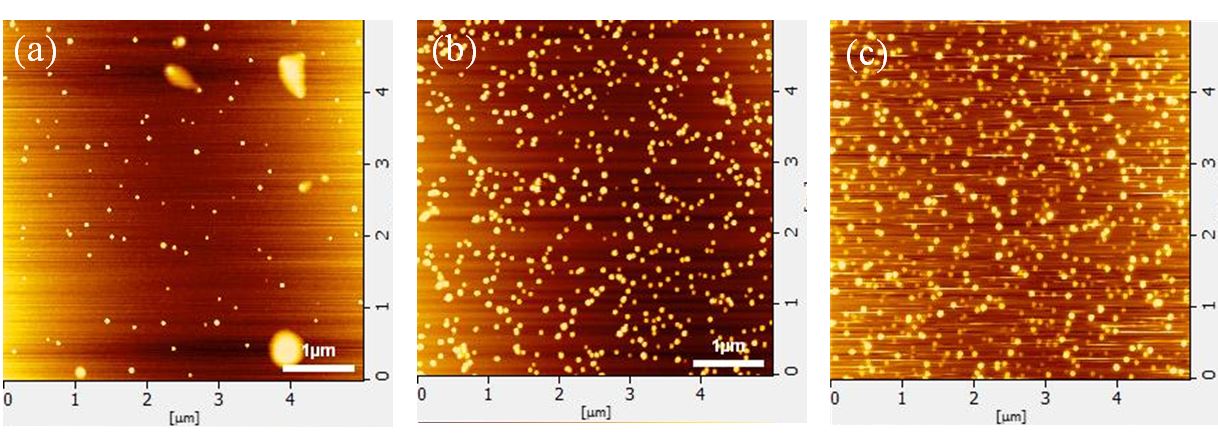


**Fig. S2:** AFM images of (a) 7 nm, (b) 10 nm, and (c) 30 nm Ag-NPs

**II. Tandem organic solar cell performance with different size and concentration of Ag-NPs inserted in IML**

**
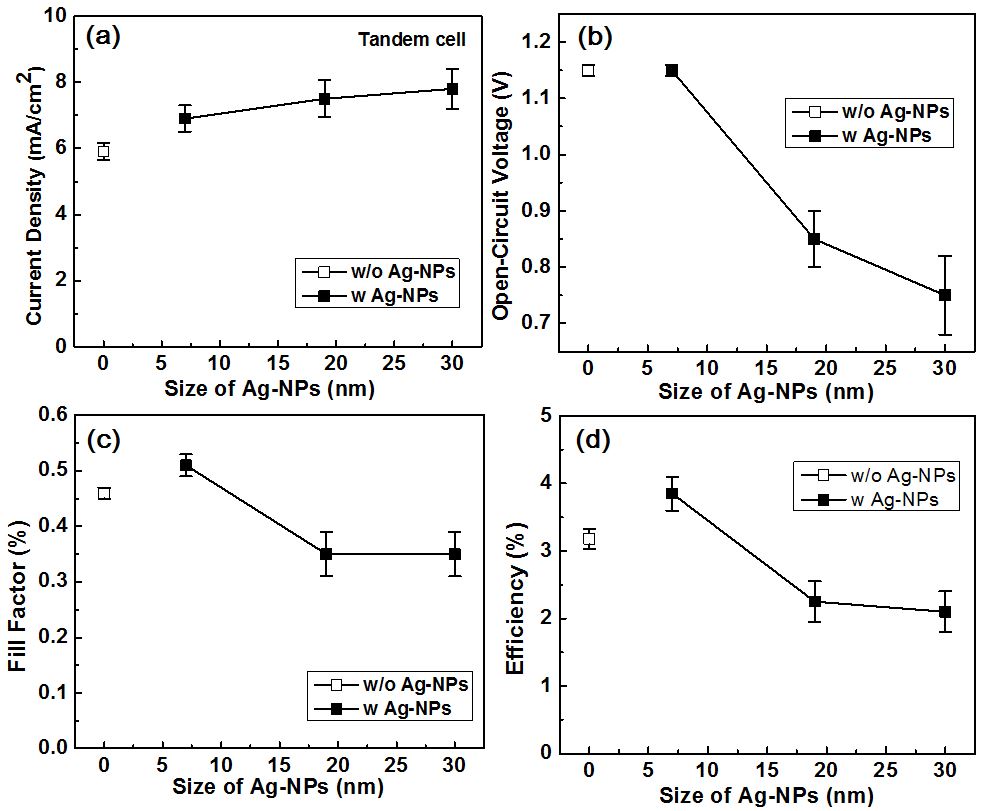
**

**Fig. S3:** Device parameters of tandem OSC according the different size of Ag-NPs, (a) Jsc, (b) Voc, (c) FF and (d) PCE


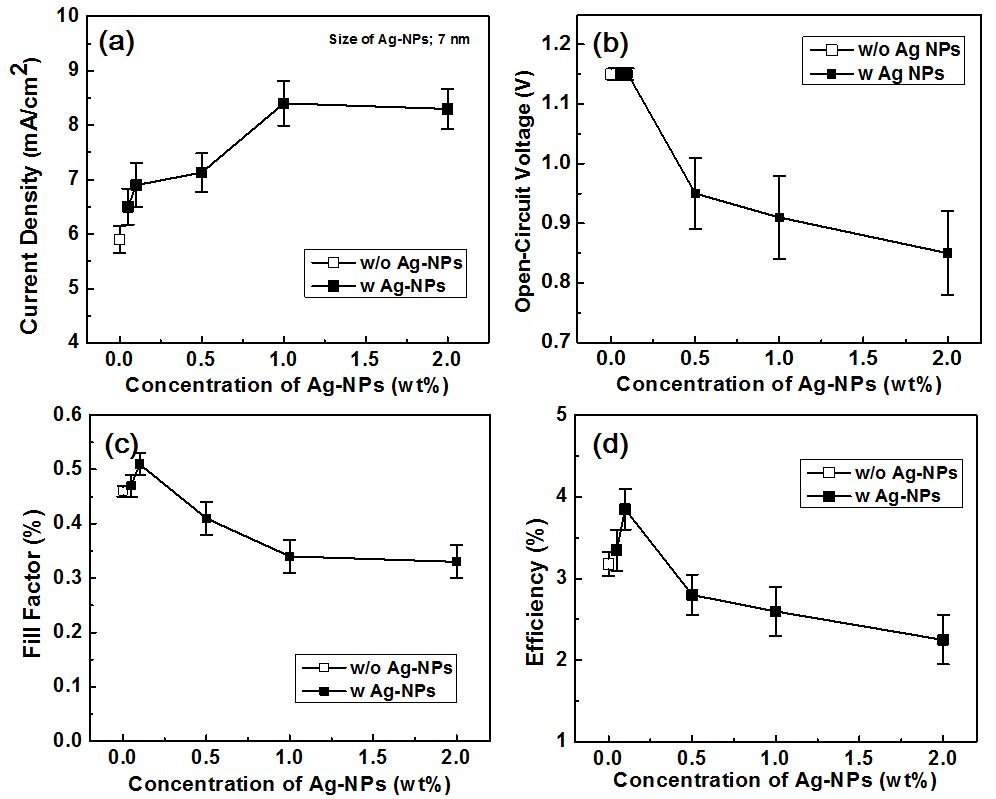


**Fig. S4:** Device parameters of tandem OSC according the concentration of 7 nm Ag-NPs, (a) Jsc, (b) Voc, (c) FF and (d) PCE

**III. Tandem organic solar cell with G-QDs inserted in IML**


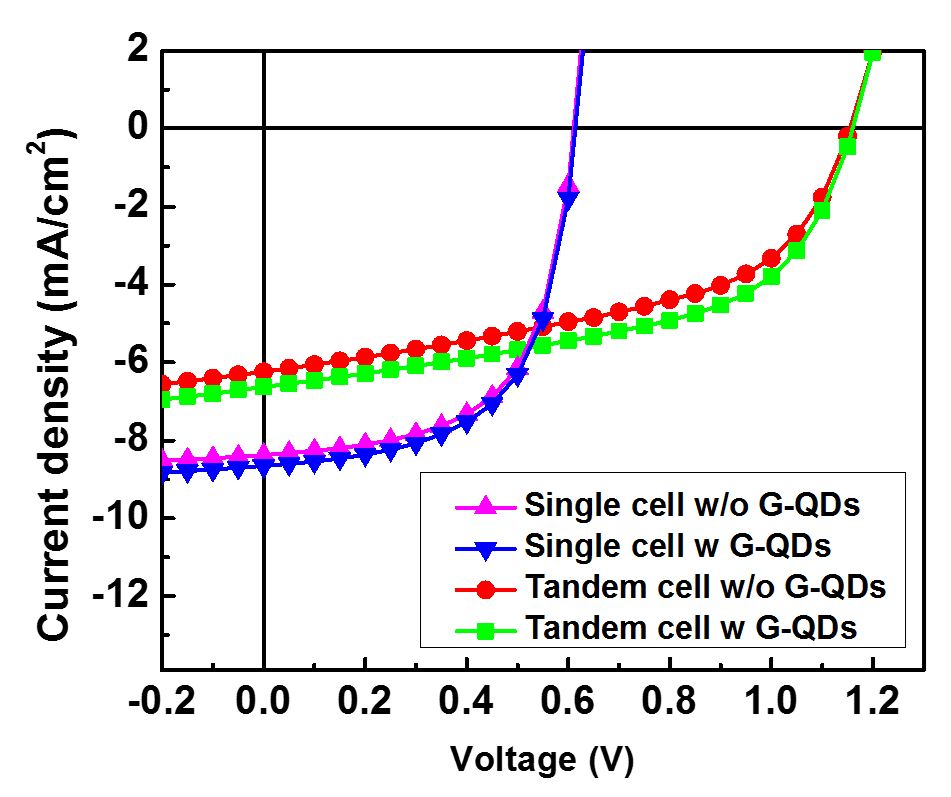


**Fig. S5:** Current density- voltage characteristic of OSC with and without G-QDs
